# Retinal organoids as models for development and diseases

**DOI:** 10.1186/s13619-021-00097-1

**Published:** 2021-11-01

**Authors:** Xiao Zhang, Wen Wang, Zi-Bing Jin

**Affiliations:** Beijing Institute of Ophthalmology, Beijing Tongren Eye Center, Beijing Tongren Hospital, Capital Medical University, Beijing Ophthalmology & Visual Science Key Laboratory, Beijing, 100730 China

## Abstract

The evolution of pluripotent stem cell-derived retinal organoids (ROs) has brought remarkable opportunities for developmental studies while also presenting new therapeutic avenues for retinal diseases. With a clear understanding of how well these models mimic native retinas, such preclinical models may be crucial tools that are widely used for the more efficient translation of studies into novel treatment strategies for retinal diseases. Genetic modifications or patient-derived ROs can allow these models to simulate the physical microenvironments of the actual disease process. However, we are currently at the beginning of the three-dimensional (3D) RO era, and a general quantitative technology for analyzing ROs derived from numerous differentiation protocols is still missing. Continued efforts to improve the efficiency and stability of differentiation, as well as understanding the disparity between the artificial retina and the native retina and advancing the current treatment strategies, will be essential in ensuring that these scientific advances can benefit patients with retinal disease. Herein, we briefly discuss RO differentiation protocols, the current applications of RO as a disease model and the treatments for retinal diseases by using RO modeling, to have a clear view of the role of current ROs in retinal development and diseases.

## Background

Over the past several decades, our knowledge of retinal diseases has significantly increased (Khan et al. 2016; Veleri et al. 2015). Despite substantial progress in the treatment of inherited retinal disease, it is still a major health problem throughout the world. One of the hurdles for translating scientific knowledge from laboratory settings to clinical settings is the lack of preclinical models. Although mouse models have significantly improved our insights into the basic concepts of retinal diseases, these models may not faithfully recapitulate pathogenic processes in patients, due to species differences (Singh et al. 2018). Currently, human pluripotent stem cell (hPSC)-derived retinal organoids (ROs) have become frequently utilized as 3D models of human retinal development and diseases, due to their easy accessibility, high stability, and proximity to native retinas (Zhang and Jin 2021).

As RO-derived retinal cells are produced in a dish, it is unlimited from a theoretical aspect. From a treatment perspective, ROs are a perfect source of retinal cells for transplantation in cell therapy and an attractive model for validating gene therapies. In recent studies, RO has also been used as a model to identify the mechanisms of inherited retinal degeneration diseases (Buskin et al. 2018; de Bruijn et al. 2020; Deng et al. 2018; Diakatou et al. 2021; Gao et al. 2020; Guo et al. 2019; Kallman et al. 2020; Kruczek et al. 2021; Lane et al. 2020; Li et al. 2019; Lukovic et al. 2020; Quinn et al. 2019; Sharma et al. 2017; Shimada et al. 2017; Zhang et al. 2020b; Zhang et al. 2021) and to test the efficiency of Adeno-associated virus (AAV) infection for retinal cells (Gonzalez-Cordero et al. 2018; Tornabene et al. 2019; Volkner et al. 2021). Additionally, RO has been used to improve the outcomes of gene therapy, to explore the membrane trafficking efficacy of some microbial opsins for cones (Garita-Hernandez et al. 2021; Garita-Hernandez et al. 2018) and to discover photoreceptor cell surface markers (Gagliardi et al. 2018; Santos-Ferreira et al. 2016) that can improve the outcomes of cell therapy. In this review, we briefly summarize the studies that have perfected the RO system, and research that using the RO as a model to enrich the knowledge about retinal development and disease; additionally, we discuss the importance of future therapeutic strategies.

## Main text

### Brief history of mouse retinal development and maturation in vivo

**Fig. 1:**
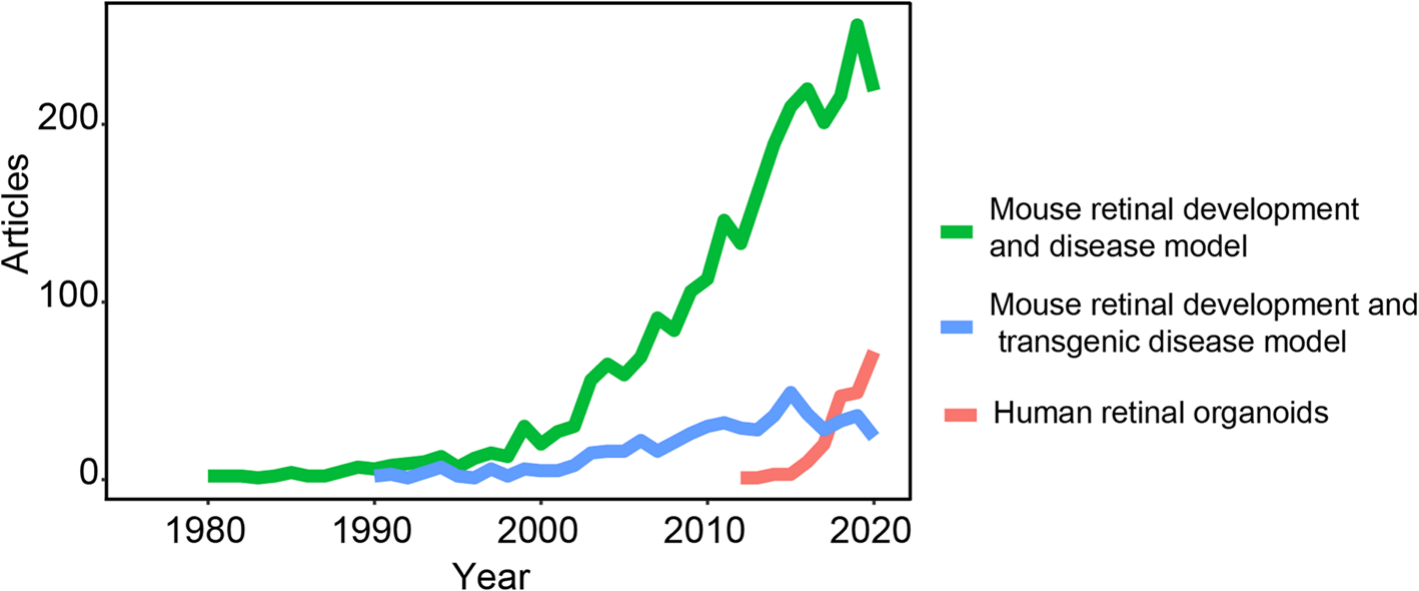
The comparison of the mouse retinal models and the human retinal organoids as models. Mouse models were used as a retinal disease model from year 1980 and transgenic mouse used to model inherited retinal disease from year 1990. In the past decades, a great increase was observed in the studies on human retinal organoids, in contrast, the studies used the mouse for retinal development models are downward

Rodent models have been the most widely used models in retina research for almost a century. These models have significantly contributed to the development, structure and function of retinal cells and their associated diseases (Keeler et al. 1928). From the year of 1980, rodent models have been used as a retinal disease model (Aguirre 1980; Albert 1980). Subsequently, the transgenic mouse disease model was developed in 1990 on retinoblastoma, which greatly encouraged mechanistic research on inherited retinal diseases for many years (Jin et al. 2014; O'Brien et al. 1990; Olsson et al. 1992; Ramamurthy et al. 2004) (Fig. 1). Due to the mouse models, we developed a complete understanding of the seven major retinal cells, including six neuronal cells (rod, cone, horizontal, amacrine, bipolar and ganglion cells) and one glial cell (Müller cell), as well as the two synaptic layers (the outer plexiform layer, OPL and the inner plexiform layer IPL) (Zhang et al. 2011). Nevertheless, the lack of human-specific features in mouse models still hampers further research on clinical applications. From 2012 onwards, 3D-cultured retinal organoids, as an emerging disease model, have become a powerful supplement to mouse models and are becoming favorable for use by scientists (Marshall and Mason 2019) (Fig. 1).

### Production of retinal organoids from human pluripotent stem cells

#### Generation of retinal cell

Photoreceptors were initially generated from hPSCs in a 2D and small-molecule induction differentiation method by the Masayo Takahashi group (Hirami et al. 2009; Osakada et al. 2009). Recently, photoreceptors have been reprogrammed from fibroblasts by using small molecules (Mahato et al. 2020). All of the molecules express specific markers and display the light response to photoreceptors, thus demonstrating an advanced degree of functionality (Hirami et al. 2009; Mahato et al. 2020; Osakada et al. 2009). The method of retinal ganglion cell (RGC) differentiation occurs at a later time than photoreceptor differentiation, which is likely due to the lack of specific RGC markers (Ohlemacher et al. 2019). It involves a stepwise differentiation method, which is characterized by a suspension culture stage with subsequent 2D adhesion culture (Ohlemacher et al. 2016; Tanaka et al. 2015). However, 2D RGCs are still combined with photoreceptors (Chen et al. 2019; Tanaka et al. 2015). Protocols for generating other retinal cells solely from human PSCs have rarely been reported. Moreover, the most common strategy for generating human retinal cells is via RO differentiation, which efficiently recapitulates the lamination structure and function of the retina (Kruczek and Swaroop 2020; Volkner et al. 2016).

#### Protocols of retinal organoid differentiation

As a model, ROs have been a part of the field for retinal development and retinal disease models for approximately 10 years. In the past decade, numerous protocols for RO differentiation have been established, based on two main approaches: 3D (Nakano et al. 2012) and a combination of 2D and 3D (Lowe et al. 2016; Zhong et al. 2014) methods. The major difference is the embryoid body (EB) stage. The 3D culture system begins with the EB stage and is followed by the optic vesicle (OV) and optic cup stages, whereas the 2D/3D culture system bypasses the EB stage and generates the OV stage from adherent retinal cells (Nakano et al. 2012; Zhong et al. 2014). The different protocols also lead to the different results and currently it is still hard to reach a consensus on the standard for the RO differentiation, in order to develop the quantitative technology for analyzing ROs, we still need time to collect more information to eliminate the effects caused by the objective and subjective variables during the RO differentiation.

There are also some modified protocols that are based on the two previously described approaches that can be summarized into three categories: chemical modification, material modification and coculture modification protocols. 1) The chemical modification protocol involves docosahexaenoic acid and fibroblast growth factor 1, which can specifically promote the maturation of photoreceptors in ROs (Brooks et al. 2019). Exogenous IGF-1 has a positive role in the RO lamination structure, and photoreceptor development relies on IGF-binding proteins (Mellough et al. 2015; Zerti et al. 2021). The combination of retinoic acid (RA), levodopa (l-DOPA), triiodothyronine (T3) and the γ-secretase inhibitor DAPT at different differentiation timepoints can lead to the direct generation of rod and cone photoreceptors in vitro (Zerti et al. 2020). Moreover, Dickkopf-related protein 1 (DKK-1), which is a Wnt signaling pathway antagonist, can induce retinal progenitors to self-organize (Luo et al. 2018). COCO, which is a multifunctional antagonist of the Wnt, TGF-β and BMP pathways, has been proven to efficiently improve the differentiation efficiency of photoreceptor precursors and cones (Pan et al. 2020; Zhou et al. 2015). Obviously, Wnt signaling plays an important role in retinal development. In addition, it significantly contributes to ocular angiogenesis, which indicates that research on Wnt signaling regulation may help in overcoming the challenges of RO vascularization (Sarin et al. 2018; Wang et al. 2019). 2) Material modifications involving bioreactors (DiStefano et al. 2018; Ovando-Roche et al. 2018) and retina-on-chip (Achberger et al. 2019; Hofer and Lutolf 2021; Manafi et al. 2021; Mittal et al. 2019), which are facilities that are used to improve the differentiation efficiency, especially retina-on-chip, which uses microfluidics to mimic the vascular energy supply. This application shows great potential in retinal disease modeling and in ophthalmology translational applications (Achberger et al. 2019; Manafi et al. 2021). 3) Coculture modification can also be used as a modified protocol. Although chemical modification and material modification can somehow enhance the RO differentiation efficiency and promote the stratified RO structure and maturation of photoreceptors, some physical cell-cell interactions are still missing in vivo, such as the RPE-photoreceptor interface, RPE-Bruch’s-choriocapillaris interactions and vascular elements, as well as microglia. The coculture system has achieved the RPE-photoreceptor interface (Achberger et al. 2019; Akhtar et al. 2019) and the RPE-Bruch’s-choriocapillaris interaction (Manian et al. 2021). However, these interfaces are only partially connected, and the integration of the RO with RPE and Bruch’s choriocapillaris, as well as microglia, will be a future area of study for regeneration medicine (Ghareeb et al. 2020).

### RO in retinal development

#### How the RO recapitulates the human in vivo retina

RO has the capability to generate the seven main major retinal cells (Collin et al. 2019; Freude et al. 2020; Kuwahara et al. 2015; Nakano et al. 2012; Zhong et al. 2014) and recapitulate retinogenesis (Mao et al. 2019; Volkner et al. 2016). Recently, the transcriptome profiling of RO and the human retina has displayed considerable similarity (Cowan et al. 2020; Sridhar et al. 2020). The cell types, specific cell differentiation markers, retinal disease genes and alternative mRNA splicing of maturing ROs are consistent with those of human retinas (Collin et al. 2019; Cowan et al. 2020; Kim et al. 2019; Mao et al. 2019; Sridhar et al. 2020). Interestingly, the cones from retinal organoids have transcriptomes that are similar to those of the human macula (Kim et al. 2019); however, the RO presents a relatively inferior inner retina lamination than the human retinas (Sridhar et al. 2020). Taken together, the retinal cell type of RO is the same as that of human retinas, but the proportions of the seven retinal cells are still different from those of human retinas. Moreover, there are more results observed on the outer retina, especially on the photoreceptors. Currently, the structure of the out retina is more similar to the native retina than the inner retina and further studies on the inner retina is necessary to perfect the retinal organoid models.

#### Seven major retinal cells in the human retinal organoids

Photoreceptors (cones and rods) are the retinal cells in ROs that possess the most details. The outer segments connected cilia and inner segments are all well displayed via transmission electron microscopy (Capowski et al. 2019; Mellough et al. 2015) and specific marker expression studies (Nakano et al. 2012; Pan et al. 2020). The typical discs of the outer segments, which are regulated by the photoreceptor cilium actin regulator C2orf71/PCARE and complex activator WASF3 (Corral-Serrano et al. 2020), also become a feature of the mature photoreceptors in ROs. In addition, the cell surface antigen CD73 has been demonstrated to be a marker for transplantable photoreceptors (Gagliardi et al. 2018; Santos-Ferreira et al. 2016). Research on rods and cones is also an interesting process. For example, rods are isolated by using real-time deformability cytometry with unique mechanical and morphological features during development, rather than isolation with the use of labels (Santos-Ferreira et al. 2019). Furthermore, thyroid hormone is identified as being a main regulator of the formation of the S-cone or M/L-cone, low levels of thyroid hormone signaling at the early stages of development can promote the S fate, and high levels of thyroid hormone signaling at late stages can promote the L/M fate (Eldred et al. 2018). The data for horizontal, amacrine and bipolar cells are relatively less than those for photoreceptors in ROs, with results only being demonstrated in cell identification during human RO differentiation (Cowan et al. 2020; Nakano et al. 2012; Zhong et al. 2014). Studies on differentiated RGCs are confluent; they can be directly induced into RGCs from retinal progenitor cells (Chavali et al. 2020; Zhang et al. 2020a) or generated as retinal cells in ROs by using the 3D/2D stepwise differentiation protocol (Chen et al. 2019; Fligor et al. 2018; Freude et al. 2020; Kobayashi et al. 2018; Rabesandratana et al. 2020), or purified from ROs (Fligor et al. 2021). RGCs in those methods all display neurite outgrowth (Chavali et al. 2020; Fligor et al. 2018; Fligor et al. 2021). Notably, Müller cells (more specifically, the factors released by Müller cells) play an important role in PSC-derived RGC survival and neuritogenesis (Pereiro et al. 2020). Early RGCs and RGCs cells can be isolated via the cell surface markers CD90 and CD171, Müller cells can be isolated via the cell surface markers CD44 and CD117, which can broadly benefit cell replacement therapies related to RGCs and Müller cells (Aparicio et al. 2017; Chavali et al. 2020; Freude et al. 2020; Shinoe et al. 2010; Too et al. 2017) (Table 1).

**Table 1:** Known cell surface markers for three retinal cells

Retinal cells	Cell surface markers	References
Photoreceptors	CD73	(Gagliardi et al. 2018; Santos-Ferreira et al. 2016)
Müller cells	CD44	(Shinoe et al. 2010; Too et al. 2017)
Early RGC	CD184/CD171	(Aparicio et al. 2017)
RGC	CD90/CD117	(Chavali et al. 2020; Freude et al. 2020)

#### Two synaptic layers in the human retinal organoids

**Fig. 2:**
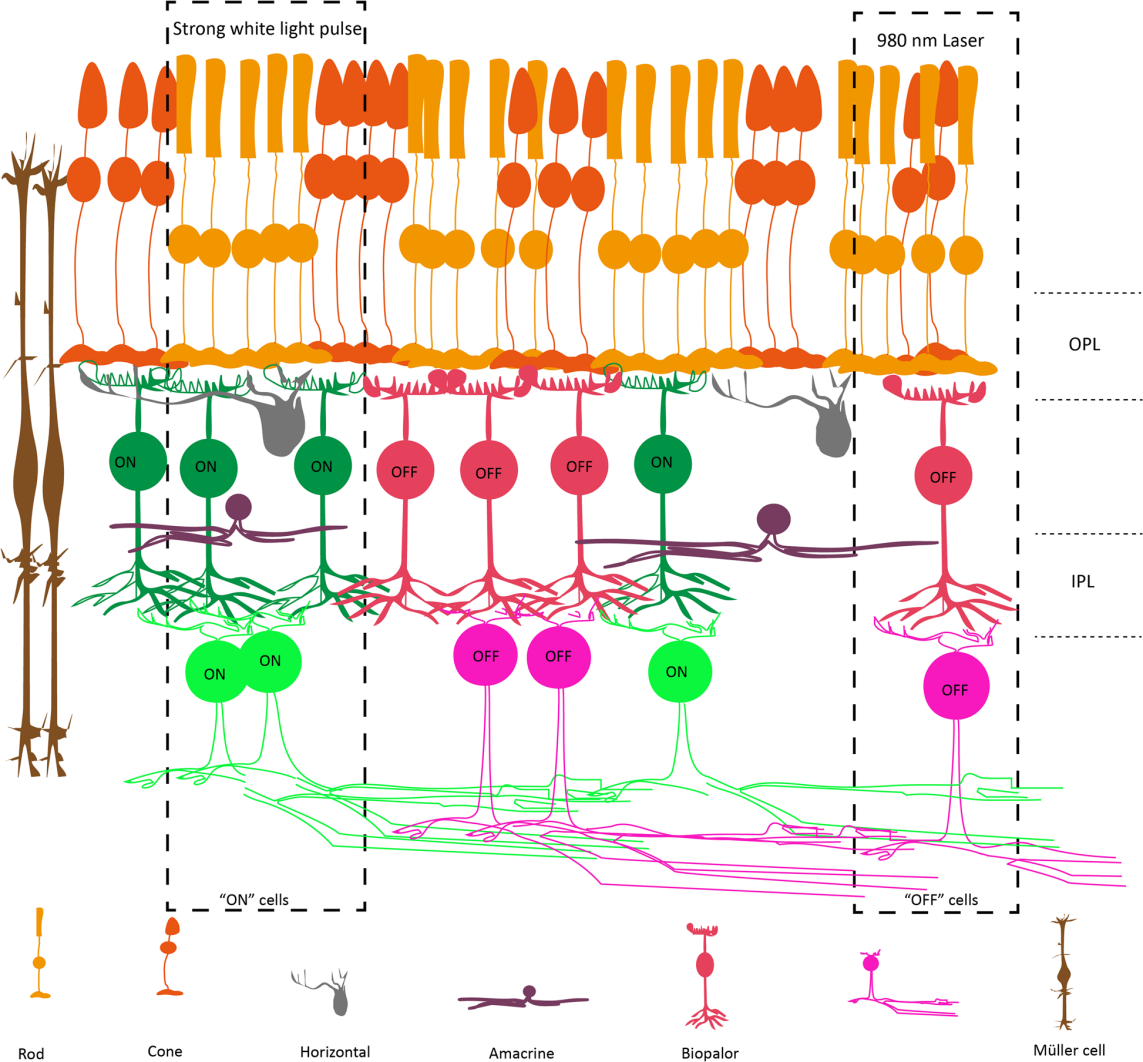
Seven retinal cells and two synaptic layers in RO. “OFF” cells are recorded at a 980 nm laser two-photon imaging (Cowan et al. 2020). “ON” cells are recorded under strong white light pulse stimuli (Dorgau et al. 2019)

The two synaptic layers contain photoreceptors that synapse onto bipolar cells, and the bipolar cells synapse onto ganglion cells. It is well known that there are OFF and ON bipolar cells, with these cells synapsing on OFF-center and ON-center ganglion cells, respectively, in the retina. Remarkably, ROs at day 150 not only form synapses, but also exhibit a phototransduction cascade and synaptic circuitry (Cowan et al. 2020; Dorgau et al. 2019; Hallam et al. 2018). Repeatable light responses can also be detected in the outer nuclear layer, 12% inner nuclear layer and RGC layers (Cowan et al. 2020; Kim et al. 2019). Additionally, these photoreceptors hyperpolarize in response to light, which indicates that recorded inner organoid cells are “OFF” cells (Cowan et al. 2020). Another study showed that the ON responses of RGCs can display a 25% increased response in spiking activity (Dorgau et al. 2019). In conclusion, the 150-day RO has been shown to generate synaptic connections in these two synaptic layers and to contain rudimentary functional synapses (Fig. 2).

### Human RO as models for disease

#### RO as an inherited retina disease model

Human PSC-derived RO models are powerful tools for identifying disease mechanisms and for developing new therapies. To date, 24 studies have used patient-derived or gene-edited human PSC-derived ROs to model inherited retinal diseases (Table 2). Twelve of these studies include retinitis pigmentosa (RP) models, which involved 11 genes (Buskin et al. 2018; de Bruijn et al. 2020; Deng et al. 2018; Diakatou et al. 2021; Gao et al. 2020; Guo et al. 2019; Kallman et al. 2020; Lane et al. 2020; Quinn et al. 2019; Sharma et al. 2017; Zhang et al. 2020b; Zhang et al. 2021). ROs have also been used to identify the disease mechanisms of Leber’s congenital amaurosis (LCA)-related *RPE65* (Li et al. 2019), *CEP290 (*Shimada et al. 2017*)*, *AIPL1* (Lukovic et al. 2020) and *CRX* (Kruczek et al. 2021). Other ocular diseases, such as glaucoma (VanderWall et al. 2020), macular telangiectasia type 2 (Gantner et al. 2019), microphthalmia (Eintracht et al. 2020), retinoblastoma (Deng et al. 2020; Liu et al. 2020; Saengwimol et al. 2020), Stargardt disease (Khan et al. 2020) and *RS1-*related X-linked juvenile retinoschisis as a model, moreover, a study on syndrome coronavirus 2 (SARS-CoV-2) demonstrated that SARS-CoV-2 can infect the retinal cells (Ahmad Mulyadi Lai et al. 2021). All these studies have been conducted in the last 4 years and have increased over time, which highlights the potential of RO as a model for retinal development and diseases. It is possible that future RO technology will enable many advances in retinal research.

**Table 2:** Summarization of the ocular disease models using ROs

Disease	Genes	Mutations	Year	Notes	References
RP	RPGR	c.1685_1686delAT	2018	Gene correct	(Deng et al. 2018)
		c.2234_2235delGA			
		c.2403_2404delAG			
RP	USH2A	c.8559-2A > G	2019		(Guo et al. 2019)
		c.9127_9129delTCC			
RP	RP2	RP2 NULL	2020	AAV gene augmentation	(Lane et al. 2020)
		RP2 R120X			
RP	CRB1	Y631C; G850S	2020		(Zhang et al. 2020b)
RP	NRL	NRL NULL	2020		(Kallman et al. 2020)
RP	PRPF31	c.1115_1125del11	2018	Gene correct	(Buskin et al. 2018)
		c.522_527 + 10del			
RP	CRB1	Homozygous M1041T	2019		(Quinn et al. 2019)
		E995X;Y631C			
		C948Y;M1041T			
RP	TRNT1	Glu43del3 GAA;p.Ser418del1Afs	2017		(Sharma et al. 2017)
		Ser418Ins1Afs; c.609-26 T > C			
RP	PDE6B	E232K	2020		(Gao et al. 2020)
RP	CLN3	A59T	2021	Gene correct	(Zhang et al. 2021)
RP	NR2E3	G56R	2021		(Diakatou et al. 2021)
RP	RP17	Structural variants	2020		(de Bruijn et al. 2020)
LCA	RPE65	L67R; Y144H	2019		(Li et al. 2019)
LCA	CEP290	Homozygous IVS26 + 1655A > G	2017		(Shimada et al. 2017)
LCA	AIPL1	C89R	2020		(Lukovic et al. 2020)
LCA	CRX	I138fs48; K88N	2021	AAV-mediated augmentation	(Kruczek et al. 2021)
Glaucoma	OPTN	E50K	2020		(VanderWall et al. 2020)
Macular telangiectasia type 2	SPTLC1	C133Y	2019		(Gantner et al. 2019)
Microphthalmia	VSX2	Homozygous R200Q	2020		(Eintracht et al. 2020)
Retinoblastoma	MYCNOS	*MYCNOS* NULL	2020		(Saengwimol et al. 2020)
Retinoblastoma	RB1	*RB1* NULL	2020		(Liu et al. 2020)
		Homozygous R320X			
Retinoblastoma	RB1	*RB1* NULL	2020		(Deng et al. 2020)
Stargardt Disease	ABCA4	c.5196 + 1137G > A	2020	Antisense oligonucleotides therapy	(Khan et al. 2020)
X-linked juvenile retinoschisis	RS1	C625T	2019	Base editing	(Huang et al. 2019)

#### RO in retinal disease therapy

As RO differentiation is convenient and stable, we can theoretically generate unlimited ROs, as well as transplantable retinal cells (Rabesandratana et al. 2020; Zhu et al. 2018). Additionally, the retinal cells in the RO have a similar stratified structure and cell-cell connection compared to the in vivo retina. In addition, ROs can either be produced from patient-derived induced PSCs (iPSCs) or from commercial human PSC lines and are more physiologically relevant than animal models. Therefore, ROs have become a reliable resource for cell therapy (Fischer et al. 2014; Gagliardi et al. 2018; Iraha et al. 2018; Kobayashi et al. 2018; McLelland et al. 2018; Singh et al. 2019; Xian et al. 2019; Xu et al. 2015; Zou et al. 2019) and drug screening (Khan et al. 2020; Liu et al. 2020), as well as a desired model for preclinical gene therapy(Garita-Hernandez et al. 2020; Gonzalez-Cordero et al. 2018; Tornabene et al. 2019; Volkner et al. 2021).

##### Cell therapy

Essentially, there are two strategies for RO-based cell therapy: transplantation of the RO specifically, of the retinal tissue (Iraha et al. 2018; McLelland et al. 2018; Singh et al. 2019), or transplantation of specific retinal cells (Gagliardi et al. 2018; Kobayashi et al. 2018; Zou et al. 2019). The major drawback of the former strategy involves integration issues. Although the transplanted retinal tissue can partially integrate with the host and display rescued retinal function, such as the light response, we can still clearly observe the boundaries between the extrinsic and intrinsic cells (Iraha et al. 2018; McLelland et al. 2018; Singh et al. 2019), which may lead to serious immune rejection without immunodeficient animal models (Iraha et al. 2018; Xian et al. 2019).

For specific retinal cell transplantation (except RPE transplantation, due to the fact that it is not directly derived from 3D-differentiated ROs), photoreceptors and retinal progenitor cells has been used for preclinical cell transplantation (Singh et al. 2020). The main problem of the retinal cell transplantation involves cell survival before and after transplantation. Before transplantation, the process of single-cell purification can lead to the deaths of retinal cells. Two-step immunopanning (Kobayashi et al. 2018) and specific cell surface markers for cell sorting (Gagliardi et al. 2018) have been shown to improve the cell survival rate (Table 1). Immune rejection also exists in this method; however, it is not as severe as tissue transplantation. Microglial activation and inflammation are crucial for the survivals of transplanted cells and host retinal cells during retinal degeneration, and the suppression of microglial activation can ameliorate the microenvironment to protect transplanted retinal cells from compromising cell viability (Zou et al. 2019). However, reactive microglia can also promote the formation of Müller-derived retinal progenitors (Fischer et al. 2014) and can facilitate retinal progenitor proliferation and differentiation into neuron-like cells (Xu et al. 2015). Therefore, modulating microglial activity is a potential approach to facilitate the success of retinal cell transplantations. Coculturing microglia with ROs and the subsequent transplantation of the microglia with retinal cells are worth performing in future studies.

##### Gene therapy

The success of AAV-mediated RPE65 gene augmentation therapy encourages the application and research on retinal diseases. The prerequisite for AAV-mediated gene therapy involves the efficient infections of specific target cells (Juttner et al. 2019) and long-lasting expressions (Garita-Hernandez et al. 2020). As an in vitro model with lamination structures and partial functions, ROs can be utilized for AAV efficiency (Gonzalez-Cordero et al. 2018; Tornabene et al. 2019; Volkner et al. 2021) and for expression time tests(Garita-Hernandez et al. 2020), which greatly enhanced transduction efficiency in the retinal cells. The promotor of AAV is the key for specifying the targeting cell. By using the human *CRX* promoter, a AAV2-*CRX* vector can precisely target a distinct apical lamina of the photoreceptor layer in the RO and function properly after infection (Kruczek et al. 2021). Thus, the effectiveness of AAV-mediated gene therapy will be significantly improved. In addition to the viral gene delivery system, the non-viral vectors based on cationic niosomes (Gallego et al. 2019; Mashal et al. 2019) and lipid nanoparticles (Patel et al. 2019) have been utilized in the rodents, the non-viral vector gene delivery system shows great potentials in therapeutic modality, for its low immunogenicity and high packing DNA size. Applying the non-viral vector to the retina organoids, is also worth trying.

The other gene therapy strategy involves gene editing of the PSC and subsequent differentiation into the RO. There have been four studies using this approach on patient-derived iPSCs (Buskin et al. 2018; Deng et al. 2018; Huang et al. 2019; Zhang et al. 2021). All of these studies proved that correct gene expression in the iPSCs can rescue the dysfunction caused by mutations in ROs. The difference among these studies includes the methods for gene editing, which involved double-strand break-based homology-directed repair in three researches (Buskin et al. 2018; Deng et al. 2018; Zhang et al. 2021) and targeted nucleotide alterations named base editing in one study (Huang et al. 2019). Although both methods exhibit off-target risks, with the accelerated progression of genome-editing technologies, it is possible that reductions in off-target editing activity (with the simultaneous maintenance of on-target editing efficiency) will be realized.

## Conclusions

Retinal organoids provide optimal platforms for modeling retinal diseases and development. More importantly, via large-scale transcriptome analyses, we are able to address the similarities and differences between the human RO and the human retina to determine how well the present RO models are recapitulating the human retina (Collin et al. 2019; Cowan et al. 2020; Kim et al. 2019; Mao et al. 2019; Sridhar et al. 2020). Additionally, ROs can reproduce the main retinal cells with correct stratified layers and can generate functional synapses. Furthermore, RO methods can supply abundant cell resources for cell therapy and provide patient derived ROs for gene therapy. So far, more than 20 patient-derived RO disease models have been established, which will ultimately provide widespread benefits and promote the development of personalized medicine.

The progression from the initial 3D retinal organoid generation and characterization of retinal cells to their development into precise disease model agents has occurred at an accelerated pace. Before the development of ROs, transgenic mice were the main models for studies on retinal development and retinal diseases. In less than 10 years, numerous RO differentiation protocols, bioreactors, retina-on-chip and coculture systems have been developed to improve the efficiency and reality of ROs, which brings us closer to the efficient production of desired retinal organoids that are equipped with vascular systems and immune systems. With the rapid development of the RO system that can mimic the native retina, a promising and well-characterized model for retinal development and retinal disease will be established, which can significantly replace mouse models.

## Data Availability

Not applicable.
